# Supplementary figures and images for: Comprehensive Modeling of Spinal Muscular Atrophy in Drosophila melanogaster
Source: Front Mol Neurosci. 2019 May 16;12:113. doi: 10.3389/fnmol.2019.00113 (PMC6532329; doi:10.3389/fnmol.2019.00113)

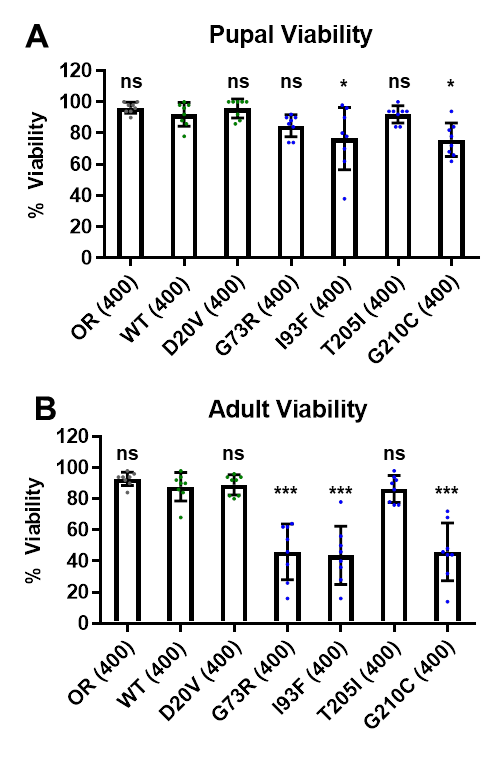

Supplement: FIGURE S1 — Survival Motor Neuron (Smn missense mutations are sufficient for viability in the absence of maternally contributed wild type SMN. (A,B) Developmental viability at the pupal stage (A) the adult stage (B) stable lines expressing only Smn missense mutations with maternal contribution of WT Smn present. Data: bars show average. Error bars show standard error. Data points represent biological replicates of 50 animals each, n-values (shown in parentheses next to genotypes) reflect the number of individual animals counted. Statistical analysis: values above the data indicate significance vs. WT from one-way ANOVA using the Dunnet correction for multiple comparisons. ns: not significant (p > 0.05), *p < 0.05, **p < 0.01, ***p < 0.001. [file Image_1.TIF]

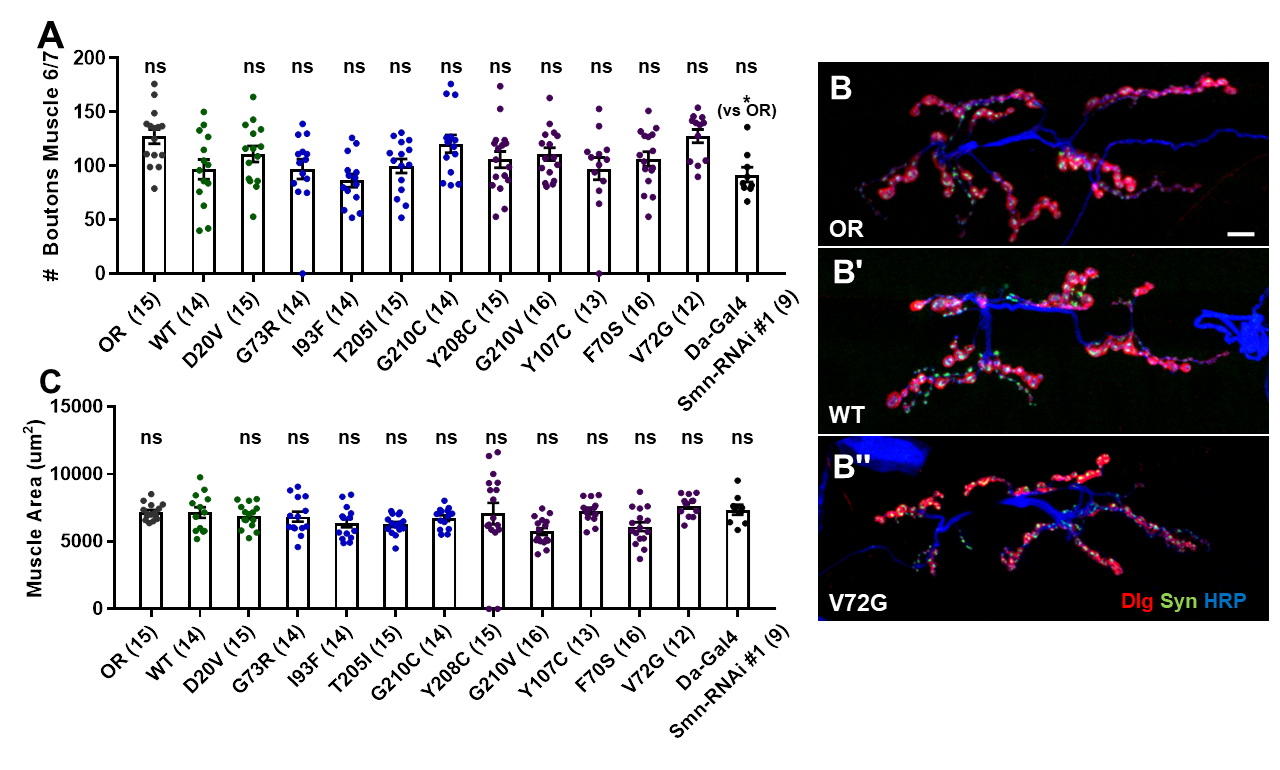

Supplement: FIGURE S2 — Survival Motor Neuron (Smn) missense mutations or knockdown has modest effects on neuromuscular junction (NMJ) structure. (A) Bouton counts from the muscle 6/7 NMJ in wandering third instar larvae. (B–B″) Representative images for the data shown in (A) for the OR (B), WT (B′), and V72G (B″) gentypes. Red marks Discs Large (Dlg), green marks synapsin (syn), and blue marks neuronal membranes. (C) Muscle 6/7 combined area for the NMJs measured in (A). Data: bars show average. Error bars show standard error. Data points and n-values (shown in parentheses next to genotypes) reflect the number of individual neuromuscular junctions (A) or muscles (C) assayed. Statistical analysis: values above the data indicate significance vs. WT from one-way ANOVA using the Dunnet correction for multiple comparisons. ns: not significant (p > 0.05), p < 0.05, p < 0.01, p < 0.001. [file Image_2.TIF]

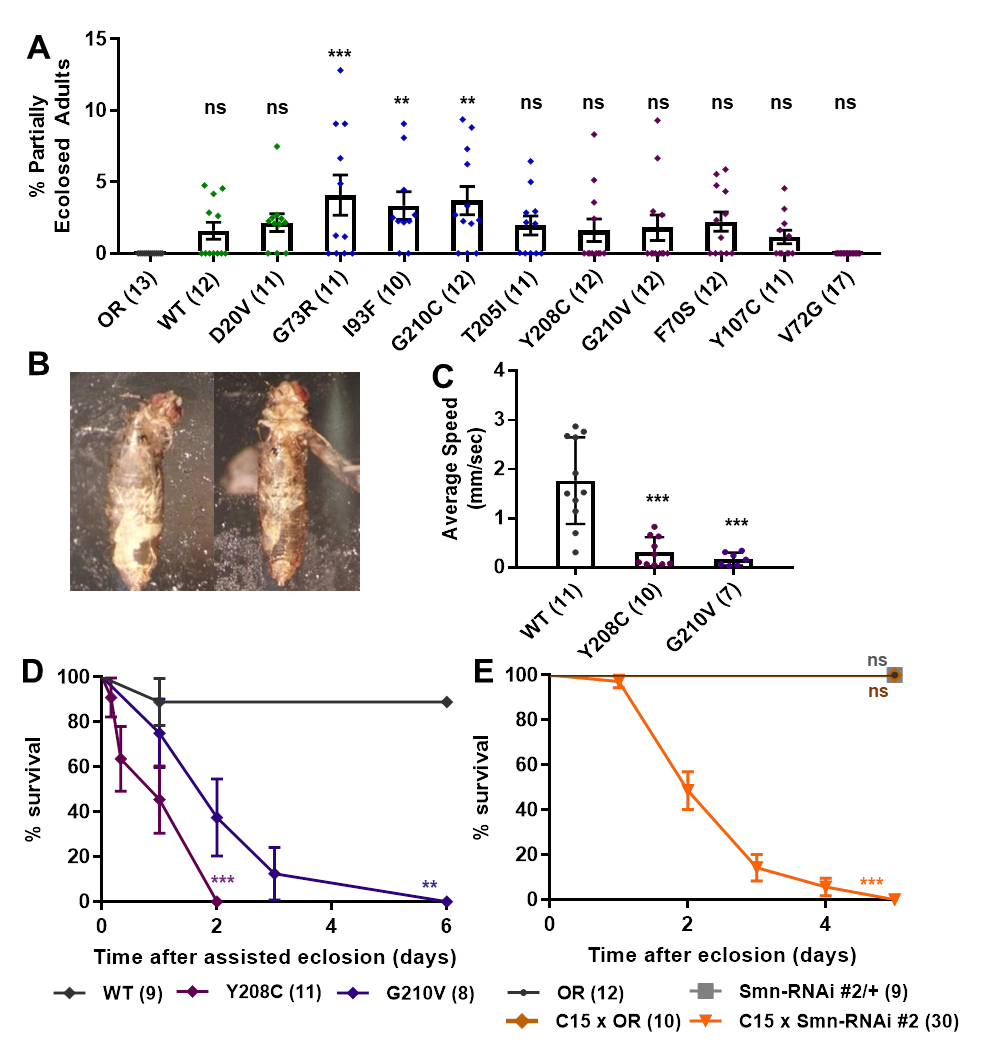

Supplement: FIGURE S3 — Smn missense mutants exhibit defects in eclosion. (A) Percent of total larvae that complete pupal development but only partially eclose from their pupal case. (B) Representative images of the partial eclosion phenotype quantified in (A). (C) Adult walking speed (mm/s) for partially eclosed animals expressing the wild type Smn transgene or the Smn missense mutations Y208C or G210V. (D) Survival curve for the same animals assayed in (C). (E) Survival curve for animals expressing neuromuscular Smn knockdown. Data: bars show average. Error bars show standard error. Data points and n-values (shown in parentheses next to genotypes) reflect the number of individual animals assayed. Statistical analysis: for (A,C), values above the data indicate significance vs. WT from one-way ANOVA using the Dunnet correction for multiple comparisons. For (D,E), values next to each survival curve represent p-values generated by Chi square analysis using the logrank rank/Mantel-Cox test. ns: not significant (p > 0.05), *p < 0.05, **p < 0.01, ***p < 0.001. [file Image_3.TIF]
