# Supplementary material for: Comprehensive Modeling of Spinal Muscular Atrophy in Drosophila melanogaster
Source: Front Mol Neurosci. 2019 May 16;12:113. doi: 10.3389/fnmol.2019.00113 (PMC6532329; doi:10.3389/fnmol.2019.00113)
Supplement: TABLE S1 — Information on all statistical analysis. Statistical analysis was performed using GraphPad Prism 7 and includes corrections for multiple comparisons when appropriate. [file Table_1.docx]

**Supplemental Table 1.** Information on all statistical analysis. (Table on next page) Statistical analysis was performed using GraphPad Prism 7 and includes corrections for multiple comparisons when appropriate.

| **Figure Panel** | **Type of Data** | **Statistical Test** | **Correction for**  **Multiple Comparison** | **F-value** | **p-value** | **R^2^** |
| --- | --- | --- | --- | --- | --- | --- |
| **1A** | **Normal** | **One-way Anova** | **Dunnet** | **F(16,120) = 24.22** | **0.0001** | **0.7635** |
| **1B** | **Normal** | **One-way Anova** | **Dunnet** | **F(16,120) = 131.7** | **0.0001** | **0.9461** |
| **1C - Early** | **Normal** | **One-way Anova** | **Dunnet** | **F(13,156) = 90.39** | **0.0001** | **0.8828** |
| **1C - Late** | **Normal** | **One-way Anova** | **Dunnet** | **F(13,156) = 32.61** | **0.0001** | **0.7321** |
| **1C - Adult** | **Normal** | **One-way Anova** | **Dunnet** | **F(13,156) = 308.6** | **0.0001** | **0.9628** |
| **Supp Fig 1A** | **Normal** | **One-way Anova** | **Dunnet** | **F(7, 56) = 8.78** | **0.0001** | **0.5232** |
| **Supp Fig 2B** | **Normal** | **One-way Anova** | **Dunnet** | **F(7, 56) = 48.93** | **0.0001** | **0.8595** |
| **2A** | **Normal** | **One-way Anova** | **Dunnet** | **F(17, 671) = 54.38** | **0.0001** | **0.5794** |
| **2C** | **Normal** | **One-way Anova** | **Dunnet** | **F(17, 671) = 44.24** | **0.00001** | **0.5303** |
| **2D** | **Normal** | **One-way Anova** | **Dunnet** | **F(13, 545) = 21.68** | **0.0001** | **0.3426** |
| **2F** | **Normal** | **One-way Anova** | **Dunnet** | **F(13, 545) = 3.545** | **0.0001** | **0.0780** |
| **Supp Fig 2A** | **Normal** | **One-way Anova** | **Dunnet** | **F(14,195) = 2.369** | **0.0046** | **0.1454** |
| **Supp Fig 2C** | **Normal** | **One-way Anova** | **Dunnet** | **F(14, 194) = 1.583** | **0.0865** | **0.1026** |
| **3D** | **Normal** | **One-way Anova** | **Dunnet** | **F(8, 55) = 16.31** | **0.0001** | **0.7035** |
| **3D** | **Normal** | **One-way Anova** | **Dunnet** | **F(8, 55) = 64.07** | **0.0001** | **0.9031** |
| **3E** | **Normal** | **One-way Anova** | **Dunnet** | **F(8, 179) = 14.03** | **0.0001** | **0.3853** |
| **4A** | **Survival data** | **Logrank/Mantel-Cox Test** | **Bonferroni** | **Corrected significance at p<0.0026** | | |
| **4B** | **Survival data** | **Logrank/Mantel-Cox Test** | **Bonferroni** | **Corrected significance at p<0.0026** | | |
| **4C** | **Survival data** | **Logrank/Mantel-Cox Test** | **Bonferroni** | **Corrected significance at p<0.0026** | | |
| **4F** | **Survival data** | **Logrank/Mantel-Cox Test** | **Bonferroni** | **Corrected significance at p<0.0056** | | |
| **4G** | **Survival data** | **Logrank/Mantel-Cox Test** | **Bonferroni** | **Corrected significance at p<0.0056** | | |
| **5A** | **Normal** | **One-way Anova** | **Dunnet** | **F(11, 1242)= 21.36** | **0.0001** | **0.1591** |
| **5C** | **Normal** | **One-way Anova** | **Dunnet** | **F(5, 157) = 9.473** | **0.0001** | **0.2318** |
| **5D** | **Normal** | **One-way Anova** | **Dunnet** | **F(5, 404) = 52.56** | **0.0001** | **39.4100** |
| **6B** | **Normal** | **One-way Anova** | **Dunnet** | **F(3, 256) = 6.607** | **0.0003** | **0.0719** |
| **6C** | **Normal** | **One-way Anova** | **Dunnet** | **F(3, 192) = 13.39** | **0.0001** | **0.1730** |
| **Supp Fig 3A** | **Normal** | **One-way Anova** | **Dunnet** | **F(13, 157)= 4.06** | **0.0001** | **0.2514** |
| **Supp Fig 3C** | **Normal** | **One-way Anova** | **Dunnet** | **F(2, 25) = 22.01** | **0.0001** | **0.6378** |
| **Supp Fig 3D** | **Survival data** | **Logrank/Mantel-Cox Test** | **Bonferroni** | **Corrected significance at p<0.025** | | |
| **Supp Fig 3E** | **Survival data** | **Logrank/Mantel-Cox Test** | **Bonferroni** | **Corrected significance at p<0.012** | | |
| **7A - 4 hrs** | **Normal** | **One-way Anova** | **Dunnet** | **F(2, 70) = 29.71** | **0.0001** | **0.4591** |
| **7A - 8 hrs** | **Normal** | **One-way Anova** | **Dunnet** | **F(2, 67) = 45.55** | **0.0001** | **0.5762** |
| **7A -12 hrs** | **Normal** | **One-way Anova** | **Dunnet** | **F(2, 73) = 62.00** | **0.0001** | **0.6294** |
| **7A - 1 day** | **Normal** | **One-way Anova** | **Dunnet** | **F(2, 73) = 97.65** | **0.0001** | **0.7279** |
| **7A - 2 days** | **Normal** | **One-way Anova** | **Dunnet** | **F(2, 73) = 129** | **0.0001** | **0.7795** |
| **7A - 3 days** | **Normal** | **One-way Anova** | **Dunnet** | **F(2, 79) = 156.6** | **0.0001** | **0.7988** |
| **7A - 4 days** | **Normal** | **Student's T-test** | **N/A** | **F(23, 24) = 1.98** | **0.0001** | **0.7659** |
| **7A -5 days** | **Normal** | **Student's T-test** | **N/A** | **F(23, 24) = 1.96** | **0.0001** | **0.7679** |
| **7A - 6 days** | **Normal** | **Student's T-test** | **N/A** | **F(23, 24) = 1.99** | **0.0001** | **0.9233** |
| **7E - 8 hrs** | **Normal** | **Student's T-test** | **N/A** | **F(89, 35) = 1.99** | **0.0001** | **0.3959** |
| **7E - 24 hrs** | **Normal** | **Student's T-test** | **N/A** | **F(89, 35) = 1.99** | **0.0001** | **0.2085** |
| **7E - 48 hrs** | **Normal** | **Student's T-test** | **N/A** | **F(89, 35) = 1.99** | **0.0001** | **0.5020** |
| **7E - 72 hrs** | **Normal** | **Student's T-test** | **N/A** | **F(89, 35) = 1.99** | **0.0001** | **0.6336** |
| **7E - 96 hrs** | **Normal** | **Student's T-test** | **N/A** | **F(89, 35) = 1.99** | **0.0001** | **0.6336** |

**Supplemental Table 2. SMN missense mutation information from human patients and fly.** When multiple SMA types and *SMN2* copy numbers are present, the order of the information for each criterion corresponds such that the first SMA type shown corresponds to the first *SMN2* copy number listed for a given mutation and so on and forth.

| **Human**  **Mutation** | **Human Protein** | **Fly**  **Protein** | **Protein**  **Domain** | **# Patients Reported** | **Human**  **SMA Type** | **SMN2 Copy #** | **Fly Phenotypic Class** | **Primary**  **Reference** |
| --- | --- | --- | --- | --- | --- | --- | --- | --- |
| **c.131A>T** | **D44V** | **D20V** | **Gemin2 binding** | **1** | **III** | **1** | **IV** | **Sun et al., 2005** |
| **c.275G>C** | **W92S** | **F70S** | **Tudor domain** | **2** | **I** | **3** | **II** | **Kotani et al., 2007** |
| **c.281T>G** | **V94G** | **V72G** | **Tudor domain** | **1** | **II** | **3** | **II** | **Clermont et al., 2004** |
| **c.283G>C** | **G95R** | **G73R** | **Tudor domain** | **1** | **III** | **1** | **III** | **Sun et al., 2005** |
| **c.346A>T** | **I116F** | **I93F** | **Tudor domain** | **1** | **I** | **1** | **III** | **Cuscó et al., 2004** |
| **c.389A>G** | **Y130C** | **Y107C** | **Tudor domain** | **1** | **NR** | **NR** | **II** | **Prior, 2007** |
| **c.788T>G** | **M263R** | **M194R** | **YG box** | **1** | **I** | **2** | **I** | **Clermont et al., 2004** |
| **c.815A>G** | **Y272C** | **Y203C** | **YG box** | **11** | **I/II/III** | **1/2/3** | **I** | **Wirth et al., 1999** |
| **c.821C>T** | **T274I** | **T205I** | **YG box** | **4** | **II/III** | **1/2** | **III** | **Wirth et al., 1999** |
| **c.856G>C** | **G275S** | **G206S** | **YG box** | **1** | **III** | **NR** | **I** | **Skordis et al 2001** |
| **c.830A>G** | **Y277C** | **Y208C** | **YG box** | **1** | **II** | **1** | **II** | **Yamamoto et al. 2013** |
| **c.868G>T** | **G279C** | **G210C** | **YG box** | **2** | **II/III** | **NR** | **III** | **Wang et al., 1998** |
| **c.869G>T** | **G279V** | **G210V** | **YG box** | **2** | **I** | **NR** | **II** | **Talbot et al., 1997** |

**Supplementary References**

Clermont, O., Burlet, P., Benit, P., Chanterau, D., Saugier-Veber, P., Munnich, A., et al. (2004). Molecular analysis of SMA patients without homozygous *SMN1* deletions using a new strategy for identification of *SMN1* subtle mutations. *Hum. Mutat.* 24, 417–427. doi: 10.1002/humu.20092

Cuscó, I., Barceló, M. J., del Río, E., Baiget, M., and Tizzano, E. F. (2004). Detection of novel mutations in the SMN Tudor domain in type I SMA patients. *Neurology* 63, 146–149. doi: 10.1212/01.wnl.0000132634.48815.13

Kotani, T., Sutomo, R., Sasongko, T. H., Sadewa, A. H., Gunadi, Minato, T., et al. (2007). A novel mutation at the N-terminal of SMN Tudor domain inhibits its interaction with target proteins. *J. Neurol.* 254, 624–630. doi: 10.1007/s00415-006-0410-x

Prior, T. W. (2007). Spinal muscular atrophy diagnostics. J. Child Neurol. 22, 952–956. doi: 10.1177/0883073807305668

Skordis, L. A., Dunckley, M. G., Burglen, L., Campbell, L., Talbot, K., Patel, S., et al. (2001). Characterisation of novel point mutations in the survival motor neuron gene SMN, in three patients with SMA. Hum. Genet. 108, 356–357. doi: 10.1007/s004390100497

Sun, Y., Grimmler, M., Schwarzer, V., Schoenen, F., Fischer, U., and Wirth, B. (2005). Molecular and functional analysis of intragenic *SMN1* mutations in patients with spinal muscular atrophy. *Hum. Mutat.* 25, 64–71. doi: 10.1002/humu.20111

Talbot, K., Ponting, C. P., Theodosiou, A. M., Rodrigues, N.R., Surtees, R., Mountford, R., Davies, K.E. (1997). Missense mutation clustering in the survival motor neuron gene: a role for a conserved tyrosine and glycine rich region of the protein in RNA metabolism? *Hum Mol Genet.* 6: 497-500.

Wang, C. H., Papendick, B. D., Bruinsma, P., and Day, J. K. (1998). Identification of a novel missense mutation of the SMNT gene in two siblings with spinal muscular atrophy. *Neurogenetics* 1, 273–276. doi: 10.1007/s100480050040

Wirth, B., Herz, M., Wetter, A., Moskau, S., Hahnen, E., Rudnik-Schoneborn, S., et al. (1999). Quantitative analysis of survival motor neuron copies: identification of subtle SMN1 mutations in patients with spinal muscular atrophy, genotype-phenotype correlation, and implications for genetic counseling. *Am. J. Hum. Genet.* 64, 1340–1356. doi: 10.1086/302369

Yamamoto, T., Sato, H., Lai, P. S., Nurputra, D. K., Harahap, N. I., Morikawa, S.,et al. (2014). Intragenic mutations in SMN1 may contribute more significantly to clinical severity than SMN2 copy numbers in some spinal muscular atrophy (SMA) patients. Brain Dev. 36, 914–920. doi: 10.1016/j.braindev.2013.11.009
